# Astaxanthin Attenuates the Changes in the Expression of MicroRNAs Involved in the Activation of Hepatic Stellate Cells

**DOI:** 10.3390/nu14050962

**Published:** 2022-02-24

**Authors:** Minkyung Bae, Mi-Bo Kim, Ji-Young Lee

**Affiliations:** 1 Department of Nutritional Sciences, University of Connecticut, Storrs, CT 06269-4017, USA; mkbae@changwon.ac.kr (M.B.); mi-bo.kim@uconn.edu (M.-B.K.); 2 Department of Food and Nutrition, Interdisciplinary Program in Senior Human Ecology, BK21 FOUR, College of Natural Sciences, Changwon National University, Changwon 51140, Korea; 3 Department of Food and Nutrition, Kyung Hee University, Seoul 02453, Korea

**Keywords:** astaxanthin, microRNA, fibrogenesis, hepatic stellate cell

## Abstract

We previously demonstrated that astaxanthin (ASTX), a xanthophyll carotenoid, has an antifibrogenic effect in hepatic stellate cells (HSC), primarily responsible for the accumulation of extracellular matrix protein during the development of liver fibrosis. Studies have shown that microRNAs (miRNAs) are involved in HSC activation. Therefore, we analyzed the expression of 84 miRNAs using miRNA arrays in primary mouse quiescent HSC (qHSC) and activated HSC (aHSC) treated with/without ASTX during their activation. Compared with qHSC, the expression of 14 miRNAs and 23 miRNAs was increased and decreased by more than 2-fold, respectively, in aHSC. Among the 14 miRNAs increased in aHSC, the expression of miR-192-5p, miR-382-5p, and miR-874-3p was reduced by ASTX. In addition, ASTX increased the expression of miR-19a-3p, miR-19b-3p, and miR-101a-3p among 23 miRNAs decreased in aHSC. Moreover, we confirmed miR-382-5p expression was ~15-fold higher in aHSC than qHSC, and ASTX markedly inhibited the induction measured by quantitative real-time PCR. We identified that the expression of *Baz1a* and *Zfp462* from the predicted miR-382-5p target genes was significantly reduced in aHSC while increased by ASTX treatment similar to the levels in qHSC. The roles of *Baz1a* and *Zfp462* in HSC activation and the antifibrogenic effect of ASTX need to be further investigated.

## 1. Introduction

Liver fibrosis is characterized by excessive accumulation of extracellular matrix (ECM) proteins, including collagen [1]. The abnormal accumulation of ECM proteins in the liver distorts the liver architecture and further impairs hepatic function [2]. Liver fibrosis occurs in most chronic liver diseases by repeated or long-lasting liver injury [3]. In developed countries, common causes of liver fibrosis include chronic hepatitis C infection, alcohol abuse, and nonalcoholic steatohepatitis (NASH) [3].

Hepatic stellate cells (HSC) play an essential role in developing liver fibrosis. In the normal liver, HSC are present in the space between endothelial cells and hepatocytes, called Space of Disse, in a quiescent state [4]. Quiescent HSC (qHSC) store vitamin A in the intracellular lipid droplets [5]. When an injury occurs in the liver, qHSC are activated and transdifferentiate into myofibroblast-like cells, i.e., activated HSC (aHSC) [4]. aHSC produce ECM proteins and inhibit their degradation by producing tissue inhibitors of metalloproteinases, leading to ECM accumulation in the liver [6].

MicroRNAs (miRNAs) are a family of endogenous short noncoding RNAs of ~21–25 nucleotides in length [7], which can regulate gene expression post-transcriptionally in a sequence-specific manner [8]. miRNAs are transcribed from miRNA genes into primary miRNAs and processed into precursor miRNAs, and finally mature miRNAs [9]. The estimated numbers of total mature miRNAs in humans and mice are 2300 [10] and 1317 [11], respectively. About 1% of mammalian genes encode miRNAs [12], and their targets are estimated to be more than 60% of mRNAs in mammals [13]. Studies have demonstrated that miRNAs are associated with human diseases, including breast cancer, lung cancer, gastric cancer, liver cancer, viral diseases, Parkinson’s disease, Alzheimer’s disease, type 2 diabetes, and nonalcoholic fatty liver disease (NAFLD) [14]. Moreover, it has been studied that miRNAs can promote HSC activation [15,16,17] or inhibit HSC activation [18,19,20,21].

Astaxanthin (ASTX) is a xanthophyll carotenoid with antioxidant properties [22]. Studies have shown that ASTX exerts antifibrogenic actions in HSC in vitro [23,24,25] and in the liver in vivo [26,27]. We previously demonstrated that ASTX inhibits HSC activation by reducing intracellular reactive oxygen species accumulation [25], decreasing the expression of histone deacetylase 9 [24], and regulating the cells’ energy metabolism [28,29]. However, the role of miRNAs in the antifibrogenic effect of ASTX in HSC has been poorly investigated. Therefore, in the present study, we sought to identify new miRNAs and their target genes that may be crucial to the antifibrogenic action of ASTX in primary mouse HSC.

## 2. Materials and Methods

### 2.1. Primary Mouse HSC Isolation and Culture

The pronase and collagenase digestion method was used to isolate primary mouse HSC from C57BL/6J mice, as previously described [23]. Primary mouse HSC were plated on uncoated plastic dishes (BD Falcon, Franklin Lakes, NJ, USA) for spontaneous activation [4] and maintained in low-glucose Dulbecco’s Modified Eagle Medium supplemented with 10% fetal bovine serum, 4 mM L-glutamine, penicillin (100 U/mL), and streptomycin (100 μg/mL) as previously described [23,25]. The cells were cultured at 37 °C under 5% CO_2_. Cell culture supplies were purchased from HyClone (Thermo Scientific, Logan, UT, USA). The cells at day 1 and day 7 after isolation represent qHSC and aHSC, respectively.

### 2.2. ASTX Treatment

ASTX was a gift kindly provided by Fuji Chemical Industry Co., Ltd. (Toyama, Japan). ASTX stock and ASTX-containing media were prepared as previously described [23]. Primary mouse HSC were treated with ASTX from day 2 to day 7 with daily media change.

### 2.3. miRNA Array and Quantitative Real-Time PCR (qRT-PCR)

Total RNA, including miRNA, was isolated from primary mouse HSC using miRNeasy Mini Kit (Qiagen, Germantown, MD, USA). Mature miRNA was selectively converted into complementary DNA (cDNA) using miScript II RT Kit (Qiagen). Mature miRNA expression profiling was measured using a pathway-focused miScript miRNA PCR Array for mouse fibrosis (Qiagen). The miRNA PCR Array layout is shown in Supplemental Figure S1. The expression of miRNAs was confirmed by qRT-PCR using miScript Primer Assays (Qiagen) in a Bio-Rad CFX96 Real-Time System (Bio-Rad, Hercules, CA, USA). All procedures were conducted according to the manufacturer’s protocols.

### 2.4. RNA Sequencing and Identification of Target Genes

RNA sequencing in primary mouse qHSC and aHSC treated with or without ASTX was conducted as previously described [28]. The miRDB [30], an online database for miRNA target prediction and functional annotations, was used for predicting the miR-382-5p target genes. Among 308 target genes of mmu-miR-382-5p predicted by miRDB, 12 potential target genes were identified based on the RNA sequencing data.

### 2.5. Target Gene Analysis by Reverse Transcription and qRT-PCR

Total RNA isolated from primary mouse qHSC and aHSC treated with or without ASTX was converted into cDNA and used for measuring the expression of target genes using the SYBR green method in a Bio-Rad CFX96 Real-Time System (Bio-Rad) as previously described [31,32].

### 2.6. Statistical Analysis

One-way analysis of variance (ANOVA) with Bonferroni correction was conducted using GraphPad Prism 6.0 (GraphPad Software, La Jolla, CA, USA). *p* values less than 0.05 were considered statistically significant. All values were expressed as mean ± standard error of the mean.

## 3. Results

### 3.1. The Expression of miRNAs Involved in Fibrosis Was Measured in qHSC, aHSC, and aHSC Treated with ASTX

**Figure 1:**
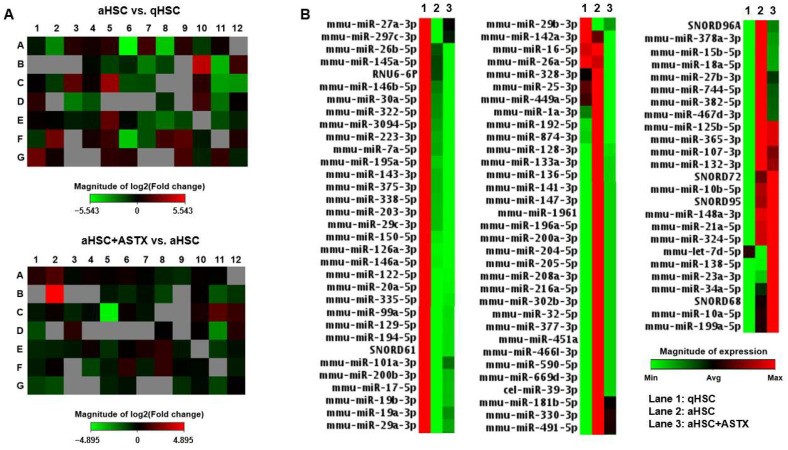
miRNA PCR array heatmaps. Primary mouse qHSC, aHSC, and aHSC treated with 25 µM of ASTX (aHSC + ASTX) were subjected to miRNA PCR array for mouse fibrosis. (**A**) Heatmaps of the miRNA array comparing miRNA profiles of aHSC vs. qHSC, and aHSC treated with ASTX (aHSC + ASTX) vs. aHSC. (**B**) Heatmap of miRNA expression in primary mouse HSC. Lanes 1, 2, and 3 show qHSC, aHSC, and aHSC + ASTX, respectively. The magnitude of expression is shown in the scale bar.

Primary mouse qHSC and aHSC treated with or without ASTX during the activation were subjected to miRNA array analysis, which can detect 84 miRNAs known to be related to fibrosis (Supplemental Figure S1). We compared the expression of miRNAs between qHSC and aHSC, and between aHSC and aHSC treated with ASTX (Figure 1A). Overall, about half of the changes in miRNA expression profiles during HSC activation were attenuated by ASTX (Figure 1B).

### 3.2. miRNAs Were Identified Whose Expression Was Altered in aHSC Compared to qHSC, Which Was Attenuated by ASTX

**Figure 2:**
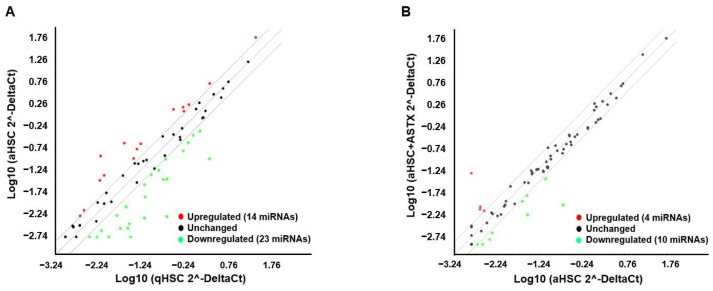
Scatter plots of miRNA expressions by miRNA PCR array. The expression of 84 miRNAs and 6 small nucleolar RNAs as log10 (2^-DeltaCt) in primary mouse aHSC and in qHSC (**A**) and in aHSC + ASTX and aHSC (**B**). The midline indicates no difference in the expression between two groups, while lines above or under the midline indicate the boundary of 2-fold regulation in the expression. Red and green dots represent each miRNA or small nucleolar RNA, upregulated and downregulated, respectively.

Among 84 miRNAs investigated, miRNAs demonstrating at least 2-fold increases or decreases during HSC activation or by ASTX treatment were selected (Figure 2A,B). There were 14 miRNAs with a more than 2-fold increase and 23 miRNAs with a more than 2-fold decrease in aHSC compared with qHSC. ASTX treatment during HSC activation upregulated 4 miRNAs and downregulated 10 miRNAs. The miRNAs whose expression was altered during HSC activation and by ASTX treatment are listed in Table 1 and Table 2.

### 3.3. ASTX Attenuated the Changes in the Expression of miRNAs during HSC Activation

**Figure 3:**
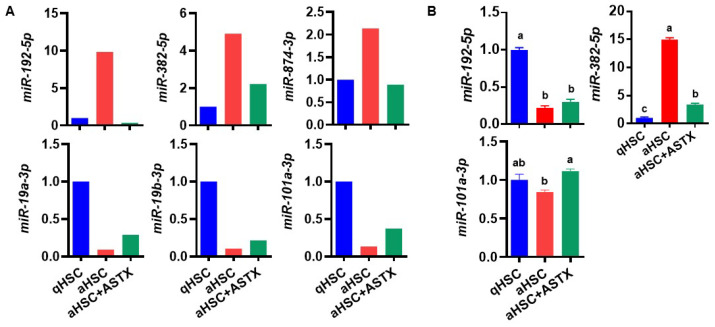
The expression of miRNAs in primary mouse HSC. (**A**) The expression of miR-192-5p, miR-382-5p, miR-874-3p, miR-19a-3p, miR-19b-3p, and miR-101a-3p from miRNA array. (**B**) The expression of miR-192-5p, miR-382-5p, and miR-101a-3p measured by qRT-PCR. *n* = 3. Bars with a different letter are significantly different (*p* < 0.05). Mean ± SEM.

To investigate whether ASTX attenuated the changes in the expression of miRNAs that were altered during HSC activation, we compared the miRNAs that showed at least 2-fold differences during HSC activation and those changed by ASTX. The expression of miR-192-5p, miR-382-5p, and miR-874-3p upregulated in aHSC was decreased by ASTX (Figure 3A). Additionally, ASTX increased the expression of miR-19a-3p, miR-19b-3p, and miR-101a-3p downregulated in aHSC.

Among those six miRNAs, miR-192-5p, miR-382-5p, and miR-101a-3p were selected for further investigation due to their high expression and higher magnitude of changes between groups. First, we confirmed their expression by qRT-PCR. The expression of miR-192-5p showed a different trend between groups from miRNA array data (Figure 3B). The expression of miR-101a-3p had a similar trend as the miRNA array result, but the magnitude of changes between groups was less than that from the array. However, the expression of miR-382-5p showed the same trend as the miRNA array with a high magnitude of changes between groups. Therefore, miR-382-5p was selected to be further investigated.

### 3.4. The Expression of Potential Target Genes of miR-382-5p Showed Drastic Differences between qHSC and aHSC, Which Were Attenuated by ASTX

**Figure 4:**
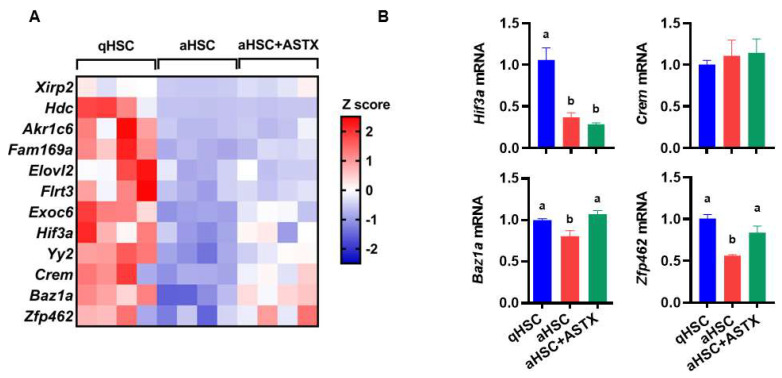
The potential target genes of miR-382-5p in primary mouse HSC. (**A**) The expression of 12 target genes of miR-382-5p was analyzed by RNA sequencing in qHSC, aHSC, and aHSC treated with ASTX (aHSC + ASTX). Z-scores for the expression are shown in the scale bar. *n* = 4. (**B**) The expression of *Hif3a*, *Crem*, *Baz1a*, and *Zfp462* measured by qRT-PCR. *n* = 6. Bars with a different letter are significantly different (*p* < 0.05). Mean ± SEM.

As miRNAs repress their target translation by inducing mRNA cleavage, mRNA degradation, or translational repression [33], the target gene expression might be reduced by miRNAs. A total of 308 potential target genes of miR-382-5p were predicted by miRDB [30]. Among the 308 target genes, 12 genes were selected whose expression was decreased during HSC activation and increased by ASTX by more than 1.5-fold based on the RNA sequencing data (Figure 4A). Potential miR-382-5p target genes include *Xirp2*, *Hdc*, *Akr1c6*, *Fam169a*, *Elovl2*, *Flrt3*, *Exoc6*, *Hif3a*, *Yy2*, *Crem*, *Baz1a*, and *Zfp462*, and their functions are listed in Table 3. We selected *Hif3a*, *Crem*, *Baz1a*, and *Zfp462* based on their known functions and expression pattern during HSC activation and ASTX treatment. We found that the expression of *Hif3a* was decreased in aHSC regardless of ASTX treatment, and *Crem* expression was not altered during HSC activation and by ASTX treatment. The expression of *Baz1a* and *Zfp462* was significantly reduced in aHSC, which was increased by ASTX to a similar level of qHSC (Figure 4B). Further studies are needed to investigate the roles of the target genes regulated by ASTX during HSC activation.

## 4. Discussion

HSC play a vital role in developing liver fibrosis as they are the primary ECM-producing cells in the liver. We previously demonstrated that ASTX attenuated the activation of HSC by decreasing the expression of fibrogenic genes [23,24,25]. To identify miRNAs that may play a crucial role in the regulation of HSC activation and be sensitive to ASTX, we performed miRNA arrays in primary mouse qHSC and aHSC treated with or without ASTX. Through our follow-up studies using RNA-Seq analysis and qRT-PCR, we identified miR-382-5p and its putative target genes, *Baz1a* and *Zfp462*, as potential mediators of the antifibrogenic effect of ASTX. As the roles of miR-382-5p, BAZ1a, and ZFP462 in HSC activation have not been studied, they may hold keys to identifying new mediators for HSC activation and expanding our understanding of how ASTX exerts an antifibrogenic effect.

Kriegel et al. [34] demonstrated that miR-382 was upregulated by transforming growth factor β (TGFβ), a potent fibrogenic cytokine, and induced during epithelial-mesenchymal transition (EMT) of human kidney epithelial cells. EMT also contributes to fibrogenesis in the liver by generating myofibroblasts [1], although its contribution may not be substantial [35]. In our previous study, ASTX prevented TGFβ1-induced fibrogenic gene expressions in human HSC line LX-2 cells [23] and primary human HSC [24]. In the present study, primary mouse aHSC had increased expression of miR-382-5p by ~15-fold compared to qHSC, which was significantly reduced by ASTX treatment. We further identified the potential target genes of miR-382-5p predicted by miRDB analyzing miRNA and target interactions [30]. Among the 308 predicted target genes, 12 genes were selected whose expression was decreased during HSC activation but increased by ASTX using RNA sequencing data. Furthermore, we selected *Hif3a*, *Crem*, *Baz1a*, and *Zfp462* based on their function and expression pattern during HSC activation and by ASTX treatment. Of these four genes, the expression of *Baz1a* and *Zfp462* was significantly reduced in aHSC, which was increased by ASTX to the level of qHSC, suggesting that these two genes may play crucial roles in mediating antifibrogenic effects of ASTX.

BAZ1A or ATP-dependent chromatin assembly factor 1 (ACF1) is an accessory, noncatalytic subunit of ACF that regulates spacing of nucleosomes using ATP to form evenly spaced nucleosomes along the chromatin [36]. The function of BAZ1A in HSC has not been reported to date. A recent study has shown that knockdown of *Baz1a* by lentivirus-mediated short hairpin RNA (shRNA) induced senescence-associated phenotypes in various cells, such as A549 (a lung adenocarcinoma cell line), U2OS (human bone osteosarcoma epithelial cells), HUVEC (human umbilical vein endothelial cells), NIH3T3 (a murine embryonic fibroblast cell line), and MEF (mouse embryo fibroblasts) [37]. The *Baz1a* knockdown upregulated genes in four signaling pathways, including p53, forkhead box O (FoxO), cell cycle, and TGFβ signaling pathways [37]. In particular, *Baz1a* knockdown increased mRNA and protein expression of SMA- and MAD-related protein 3 (SMAD3), an important mediator of the TGFβ signaling pathway [38], in A549 and U2OS cells [37]. As we previously reported that the antifibrogenic effect of ASTX was mediated by SMAD3 in HSC [23], BAZ1A may play a crucial role in HSC activation and mediate the antifibrogenic activity of ASTX in HSC. In addition, *Zfp462* expression was significantly decreased in aHSC compared to qHSC, which was inhibited by ASTX in the present study. ZFP462 or ZNF462 belongs to the C2H2-type zinc finger family of proteins, which is involved in transcription by regulating chromatin structure [39]. ZFP462 is known to be crucial for early embryonic development [40] and neuronal differentiation [41]. The role of ZFP462 in HSC activation has not been determined, and thus it is worthwhile to investigate whether ZFP462 is an important mediator of HSC activation.

In the present study, we focused on miR-382-5p due to its high expression and the magnitude of changes between groups in primary mouse HSC. The other five potential target miRNAs, including miR-192-5p, miR-874-3p, miR-19a-3p, miR-19b-3p, and miR-101a-3p, may be worthy of future investigation. Studies have demonstrated that circulating miR-192 is upregulated in patients with NAFLD [42], NASH [43], alcoholic hepatitis [44], and acetaminophen-induced liver injury [45]. Circulating miR-192 was also increased in mice fed alcohol [44] and mice with acetaminophen-induced liver injury [46]. In addition, exosomal transport of miR-192 from hepatitis C virus-infected hepatocytes increased the protein expression of fibrogenic markers, such as procollagen type I α1 and α-smooth muscle actin in LX-2 cells [47]. Moreover, miR-192 was induced by TGFβ in rat tubular epithelial cells [48] and mouse mesangial cells [49]. However, miR-192 expression is lower in primary mouse aHSC than qHSC [50], consistent with the present study. In addition, primary HSC isolated from mice with carbon tetrachloride (CCl_4_) or bile duct ligation-induced liver fibrosis showed a decrease in miR-192 expression compared with control [50]. The discrepancy between studies may result from different stages of HSC activation. In addition, the in vivo activation of HSC may cause a different result, as aHSC are exposed to various other factors from neighboring cells in vivo. Therefore, further studies are necessary to investigate the expression and function of miR-192 in the activation of HSC in human livers with various pathologies, mouse livers with liver fibrosis, or HSC.

There are limited studies examining the functions of the other four potential miRNAs, i.e., miR-874-3p, miR-19a-3p, miR-19b-3p, and miR-101a-3p, in HSC. Consistent with our findings in primary mouse HSC, miR-874 was upregulated in rat aHSC compared with qHSC [51]. However, several studies have reported that miR-874 expression was reduced in the liver of patients with hepatocellular carcinoma [52,53,54]. The expression of miR-101a was decreased in the CCl_4_-induced fibrotic mouse liver and mouse aHSC [55]. In addition, HSC-T6 cells, a rat HSC line, transfected with miR-101 showed suppressed proliferation, migration, and TGFβ signaling [55]. Our study showed no change in miR-101a expression during HSC activation, while it was increased by ASTX. It suggests that the antifibrogenic effect of ASTX may be partially mediated by the induction of miR-101a. miR-19a and miR-19b are known to be downregulated in primary rat aHSC and the fibrotic rat and human liver [56]. miR-19b inhibited transdifferentiation of primary rat HSC by reducing phosphorylated SMAD3 [56]. We previously demonstrated that ASTX attenuates TGFβ1-induced phosphorylation and nuclear translocation of SMAD3 in LX-2 cells [23]. Based on our observation, the effect of ASTX on the level of miR-19a and miR-19b was minimal; thus, the role of ASTX in the regulation of SMAD3 may not be mediated by miR-19.

There is limited, somewhat conflicting information on miRNA changes in HSC and the liver. It is probable that miRNA expression in HSC and the liver may differ depending on the stage of HSC activation and liver pathogenesis. Regardless, we demonstrate the potential role of miR-382-5p and its target genes in the activation of HSC and liver diseases. Therefore, future investigation is warranted to gain detailed insight into their functions in regulating HSC activation in vivo and in vitro.

## 5. Conclusions

In our previous studies, we have demonstrated that ASTX has antifibrogenic properties in HSC [24,25,28,29]. The present study provides potential miRNAs, especially miR-382-5p, possibly involved in the antifibrogenic effect of ASTX during HSC activation. In addition, we identified target genes of miR-382-5p, including *Baz1a* and *Zfp462*, for further investigation to determine their roles in HSC activation. Therefore, this study provides a new avenue of investigation to dissect the mechanisms for HSC activation and the antifibrogenic effect of ASTX.

## Figures and Tables

**Table 1:** Fold changes of the expression of miRNAs in primary mouse aHSC compared to qHSC. The lists of miRNAs overexpressed and underexpressed during HSC activation.

Overexpressed	Fold Change	Underexpressed	Fold Change
miRNAs	(aHSC vs. qHSC)	miRNAs	(aHSC vs. qHSC)
mmu-miR-148a-3p	17.35	mmu-miR-122-5p	0.02
mmu-miR-192-5p	9.83	mmu-miR-126a-3p	0.05
mmu-miR-324-5p	5.23	mmu-miR-335-5p	0.05
mmu-miR-382-5p	4.91	mmu-miR-150-5p	0.07
mmu-miR-27b-3p	4.31	mmu-miR-19a-3p	0.09
mmu-miR-181b-5p	3.91	mmu-miR-19b-3p	0.11
mmu-miR-365-3p	3.7	mmu-miR-101a-3p	0.13
mmu-miR-744-5p	2.9	mmu-miR-200b-3p	0.16
mmu-miR-34a-5p	2.63	mmu-miR-146a-5p	0.16
mmu-miR-21a-5p	2.58	mmu-miR-223-3p	0.18
mmu-miR-125b-5p	2.53	mmu-miR-29b-3p	0.19
mmu-miR-15b-5p	2.44	mmu-miR-203-3p	0.29
mmu-miR-330-3p	2.32	mmu-miR-29a-3p	0.3
mmu-miR-874-3p	2.14	mmu-miR-338-5p	0.31
		mmu-miR-3094-5p	0.31
		mmu-miR-194-5p	0.32
		mmu-miR-195a-5p	0.34
		mmu-miR-29c-3p	0.35
		mmu-miR-146b-5p	0.37
		mmu-miR-143-3p	0.39
		mmu-miR-129-5p	0.43
		mmu-miR-17-5p	0.45
		mmu-miR-322-5p	0.47

**Table 2:** Fold changes of the expression of miRNAs in primary mouse aHSC treated with ASTX compared with control aHSC ^1^.

	miRNAs	Fold Change (aHSC + ASTX vs. aHSC)
Overexpressed	mmu-miR-138-5p	29.75
	mmu-miR-19a-3p	3.17
	mmu-miR-101a-3p	2.80
	mmu-miR-19b-3p	2.05
Underexpressed	mmu-miR-192-5p	0.04
	mmu-miR-223-3p	0.16
	mmu-miR-150-5p	0.33
	mmu-miR-449a-5p	0.37
	mmu-miR-1a-3p	0.37
	mmu-miR-328-3p	0.41
	mmu-miR-874-3p	0.42
	mmu-miR-146b-5p	0.43
	mmu-miR-382-5p	0.45
	mmu-miR-3094-5p	0.50

^1^ The lists of miRNAs overexpressed and underexpressed by ASTX treatment.

**Table 3:** The potential target genes of miR-382-5p altered during HSC activation and ASTX treatment in HSC.

Gene	Full Name	Function
*Xirp2*	Xin actin-binding repeat containing 2	Xirp2 belongs to muscle-specific, actin-binding Xin gene family. It is expressed in cardiac and skeletal muscle interacting with filamentous actin and α-actinin via the actin-binding motif, the Xin repeat.
*Hdc*	Histidine decarboxylase	HDC catalyzes the decarboxylation of histidine to form histamine.
*Akr1c6*	Aldo-keto reductase family 1, member C6	Akr1c6 encodes estradiol 17 β-dehydrogenase 5, which catalyzes the reduction of 4-androstenedione, 5-α-androstane-3,17-dione, androsterone and dehydroepiandrosterone to testosterone, dihydrotestosterone, 5-α-androstane-3-α,17-β-diol, and 5-androstene-3-β,17-β-diol, respectively.
*Fam169a*	Family with sequence similarity 169, member A	Soluble lamina-associated protein of 75 kD.
*Elovl2*	Elongation of very long chain fatty acids (FEN1/Elo2, SUR4/Elo3, yeast)-like 2	ELOVL2 is a condensing enzyme catalyzing the elongation of long-chain polyunsaturated fatty acids.
*Flrt3*	Fibronectin leucine rich transmembrane protein 3	FLRT3 is involved in cell–cell adhesion, cell migration, and axon guidance.
*Exoc6*	Exocyst complex component 6	EXOC6 is a component of the exocyst complex involved in vesicle trafficking, specifically the tethering of secretory vesicles to the plasma membrane during exocytosis.
*Hif3a*	Hypoxia inducible factor 3, alpha subunit	HIF3A belongs to the transcription factor family of hypoxia-inducible factors, which regulate the cellular response to hypoxia.
*Yy2*	Yy2 transcription factor	Yy2 acts as a multifunctional transcription factor regulating a large number of genes positively and negatively. It is involved in development and differentiation.
*Crem*	cAMP responsive element modulator	CREM is a component of cAMP-mediated signal transduction during various physiological processes, including spermatogenesis, cardiac function, and circadian rhythm.
*Baz1a*	Bromodomain adjacent to zinc finger domain 1A	BAZ1A is the accessory, noncatalytic subunit of the ATP-dependent chromatin assembly factor, which regulates spacing of nucleosomes using ATP to form evenly spaced nucleosomes along the chromatin.
*Zfp462*	Zinc finger protein 462	ZFP462 or ZNF462 belongs to C2H2-type zinc finger family of proteins. It is involved in transcription by regulating chromatin structure.

## Data Availability

Not applicable.

## Supplementary Materials

The following supporting information can be downloaded at: https://www.mdpi.com/article/10.3390/nu14050962/s1, Figure S1: miRNA PCR array layout for mouse fibrosis.
