# Supplementary material for: Astaxanthin Attenuates the Changes in the Expression of MicroRNAs Involved in the Activation of Hepatic Stellate Cells
Source: Nutrients. 2022 Feb 24;14(5):962. doi: 10.3390/nu14050962 (PMC8912553; doi:10.3390/nu14050962)
Supplement: Supplementary file 1 [file nutrients-14-00962-s001.zip › nutrients-1597235-supplementary.pdf]

Supplementary Materials

Figure S1. miRNA PCR array layout for mouse fibrosis. miRNA PCR array layout containing 84 miRNAs and controls, including Caenorhabditis elegans miR-39, 6 small nucleolar RNAs, miRNA reverse transcription control (miRTC), and positive PCR control (PPC).

	1	2	3	4	5	6	7	8	9	10	11	12
A	mmu-let-7d-5p	mmu-miR-101a-3p	mmu-miR-107-3p	mmu-miR-10a-5p	mmu-miR-10b-5p	mmu-miR-122-5p	mmu-miR-125b-5p	mmu-miR-126a-3p	mmu-miR-128-3p	mmu-miR-129-5p	mmu-miR-132-3p	mmu-miR-133a-3p
B	mmu-miR-136-5p	mmu-miR-138-5p	mmu-miR-141-3p	mmu-miR-142a-3p	mmu-miR-143-3p	mmu-miR-145a-5p	mmu-miR-146a-5p	mmu-miR-146b-5p	mmu-miR-147-3p	mmu-miR-148a-3p	mmu-miR-150-5p	mmu-miR-15b-5p
C	mmu-miR-16-5p	mmu-miR-17-5p	mmu-miR-181b-5p	mmu-miR-18a-5p	mmu-miR-192-5p	mmu-miR-194-5p	mmu-miR-195a-5p	mmu-miR-1961	mmu-miR-196a-5p	mmu-miR-199a-5p	mmu-miR-19a-3p	mmu-miR-19b-3p
D	mmu-miR-1a-3p	mmu-miR-200a-3p	mmu-miR-200b-3p	mmu-miR-203-3p	mmu-miR-204-5p	mmu-miR-205-5p	mmu-miR-208a-3p	mmu-miR-20a-5p	mmu-miR-216a-5p	mmu-miR-21a-5p	mmu-miR-223-3p	mmu-miR-23a-3p
E	mmu-miR-25-3p	mmu-miR-26a-5p	mmu-miR-26b-5p	mmu-miR-27a-3p	mmu-miR-27b-3p	mmu-miR-297c-3p	mmu-miR-29a-3p	mmu-miR-29b-3p	mmu-miR-29c-3p	mmu-miR-302b-3p	mmu-miR-3094-5p	mmu-miR-30a-5p
F	mmu-miR-322-5p	mmu-miR-324-5p	mmu-miR-32-5p	mmu-miR-328-3p	mmu-miR-330-3p	mmu-miR-335-5p	mmu-miR-338-5p	mmu-miR-34a-5p	mmu-miR-365-3p	mmu-miR-375-3p	mmu-miR-377-3p	mmu-miR-378a-3p
G	mmu-miR-382-5p	mmu-miR-449a-5p	mmu-miR-451a	mmu-miR-466l-3p	mmu-miR-467d-3p	mmu-miR-491-5p	mmu-miR-590-5p	mmu-miR-669d-3p	mmu-miR-744-5p	mmu-miR-7a-5p	mmu-miR-874-3p	mmu-miR-99a-5p
H	C. elegans miR-39	C. elegans miR-39	SNORD61	SNORD68	SNORD72	SNORD95	SNORD96A	RNU6-6P	miRTC	miRTC	PPC	PPC
